# Human Astrocyte Spheroids as Suitable In Vitro Screening Model to Evaluate Synthetic Cannabinoid MAM2201-Induced Effects on CNS

**DOI:** 10.3390/ijms24021421

**Published:** 2023-01-11

**Authors:** Uliana De Simone, Patrizia Pignatti, Laura Villani, Luciana Alessandra Russo, Azzurra Sargenti, Simone Bonetti, Eleonora Buscaglia, Teresa Coccini

**Affiliations:** 1 Laboratory of Clinical and Experimental Toxicology, and Pavia Poison Centre-National Toxicology Information Centre, Toxicology Unit, Istituti Clinici Scientifici Maugeri IRCCS, 27100 Pavia, Italy; 2 Allergy and Immunology Unit, Istituti Clinici Scientifici Maugeri IRCCS, 27100 Pavia, Italy; 3 Pathology Unit, Istituti Clinici Scientifici Maugeri IRCCS, 27100 Pavia, Italy; 4 CellDynamics srl, 40129 Bologna, Italy; 5 CNR-ISMN, Institute for Nanostructured Materials, 40129 Bologna, Italy

**Keywords:** CNS toxicity, in vitro 3D models, human astrocytes, preclinical studies, novel psychoactive substances, public health

## Abstract

There is growing concern about the consumption of synthetic cannabinoids (SCs), one of the largest groups of new psychoactive substances, its consequence on human health (general population and workers), and the continuous placing of new SCs on the market. Although drug-induced alterations in neuronal function remain an essential component for theories of drug addiction, accumulating evidence indicates the important role of activated astrocytes, whose essential and pleiotropic role in brain physiology and pathology is well recognized. The study aims to clarify the mechanisms of neurotoxicity induced by one of the most potent SCs, named MAM-2201 (a naphthoyl-indole derivative), by applying a novel three-dimensional (3D) cell culture model, mimicking the physiological and biochemical properties of brain tissues better than traditional two-dimensional in vitro systems. Specifically, human astrocyte spheroids, generated from the D384 astrocyte cell line, were treated with different MAM-2201 concentrations (1–30 µM) and exposure times (24–48 h). MAM-2201 affected, in a concentration- and time-dependent manner, the cell growth and viability, size and morphological structure, E-cadherin and extracellular matrix, CB1-receptors, glial fibrillary acidic protein, and caspase-3/7 activity. The findings demonstrate MAM-2201-induced cytotoxicity to astrocyte spheroids, and support the use of this human 3D cell-based model as species-specific in vitro tool suitable for the evaluation of neurotoxicity induced by other SCs.

## 1. Introduction

New psychoactive substances (NPSs) are continuously emerging onto the illicit drug market. Among the most abused NPSs, the synthetic cannabinoids (SCs) are the largest group, with 190 compounds reported to the European Monitoring Centre for Drugs and Drug Addiction [1,2] by the end of 2018.

These “Spice”, “K2”, and “herbal blend” drugs comprise different compounds that bind to the cannabinoid receptors CB1 and CB2 [3,4,5,6,7] and cause psychotropic effects similar to those of Δ9-tetrahydrocannabinol (THC) and its structural analogs [8,9]. Some of the SCs marketed so far are full agonists of the CB1 and CB2 receptors, and show a much higher affinity and potency than the natural cannabis ingredient THC, enabling stronger in vivo pharmacodynamic effects at lower doses [10,11,12,13,14].

A recent in vivo study demonstrated that the acute administration of some SCs (such as JWH-018-Cl, JWH-018-Br, and the fluorinate analog AM-2201) alters visual, acoustic, and tactile sensorimotor responses in mice [15].

The pharmacological effects of certain “Spice” drugs have been investigated by several groups, but information and publications on their cytotoxic and long-term toxic properties are still scarce.

Despite the marked differences in their chemical structure, all SCs are lipid soluble, are non-polar, and typically consist of 20–26 carbon atoms [10]. SCs can easily cross the blood–brain barrier and exert their actions on the central nervous system (CNS), causing adverse effects [16]. The main components of the “Spice” and “K2” drugs are naphthoyl-indole derivatives, which are the first-generation of SCs and elicit excitation behaviors by themselves, as reported in intoxicated patients with MAM-2201 [17,18], one of the potent cannabinoid CB1 receptor full agonists (Ki: 2.07 ± 0.82 nM) [19,20,21]. MAM-2201 has also shown a high affinity (Ki) and activation potential for human CB2 receptors, as indicated by the Ki, i.e., 0.582 ± 0.123 nM (full agonist) [19]. In humans, severe effects have been associated with MAM-2201, including death [22,23]. Yet, there is limited toxicological and mechanistic information available regarding the brain effects induced by SCs. Currently, toxicity data on SCs and in particular on MAM-2201, which has been frequently identified in herbal blends or in powder products, are mainly obtained from studies performed on in vitro rodent cells and in vivo animal models [24,25,26]. The limited experimental evidence on neuronal cultures (rodent) indicate that MAM-2201 exposure can cause impairments of neuronal function and activity, as well as the inhibition of neurotransmitter release, which are effects likely mediated by the CB1 receptor [25,27,28,29]. Moreover, abnormal behaviors have been observed in animal models [30].

Globally, there is at present no obvious universal mechanism whereby plant-derived, synthetic, and endogenous cannabinoids affect cell viability and proliferation, particularly in human brain cells. Our recent investigation evidenced a higher susceptibility (e.g., increases in cell mortality and apoptosis) on human primary neuronal cells and the astrocyte line compared to rodent cultures when treated with MAM-2201. In human neurons, cytotoxic effects and the loss of neuronal markers were detected at low concentrations, i.e., 1–5 µM, early (after 3 h) as well as after 48 h. Human astrocytes were also targeted by MAM-2201. The different altered endpoints were reversed, attenuated, or not antagonized by AM251, a selective CB1 receptor antagonist, indicating that the CB1 receptors may in part mediate MAM-2201-induced cytotoxicity in human astrocytes [31].

Although drug-induced alterations in neuronal function remain an essential component for theories of drug addiction [32], the accumulating evidence suggests that they are not the only cells impacted by drugs of abuse. Glial cells, including microglia and astrocytes, are also influenced by exposure to abused drugs (e.g., cocaine, amphetamines, morphine, and MDMA), and their responses likely contribute to the behavioral outcomes associated with substance abuse [33,34]. Astrocytes are the most abundant cell type in the CNS and have traditionally been characterized as supportive cells in the brain for their roles in maintaining neuron homeostasis and survival. They are well positioned to integrate signals from a great number of synapses at once, which may have implications for higher-order information processing [35]. Astrocyte activity has been shown to be involved in acute injury and neurodegeneration; to affect neuroplasticity, learning, and memory; and to accompany neuropsychiatric disorders, drug addictions, and drug dependence [36,37].

The role of activated astrocytes in drug addiction has recently been well recognized [38], and due to the essential and pleiotropic role of astrocytes in brain physiology and pathology, they have gained enormous interest in recent decades as a potential target for neurotherapies [39,40].

Cannabinoid compounds are known to induce psychotropic effects by activating the CB1 receptors expressed by neurons [41], but also by modifying essential central and peripheral physiological processes by activating the CB1 and CB2 receptors expressed by glial cells and peripheral cells [42]. The cannabinoid system and its ligands have been shown to interact with and affect the activities of astrocytes [39]. Neurons express mostly CB1 cannabinoid receptors, while microglia express CB2. Both types of cannabinoid receptors have been found to be present on astrocytes [39,42]. The data in this area, however, are in their infancy and are confined to the major constituents of cannabis [39,43].

Furthermore, it is recognized that astroglial type-1 cannabinoid (CB1) receptors are involved in synaptic transmissions, plasticity, and behavior by interfering with the so-called tripartite synapse formed by pre- and post-synaptic neuronal elements and surrounding astrocyte processes [44,45,46,47,48,49,50,51,52]. CBs have also been reported to participate in a variety of physiological processes, such as learning, memory, and pain sensations.

An increasing number of reports have indicated that chronic and acute stressors alter astrocyte morphology and the expression of astrocyte-specific proteins in brain areas that are known to play a critical role in emotional processing, such as the prefrontal cortex, hippocampus, and amygdala [53,54,55].

In this context, with the aim of clarifying the mechanisms of neurotoxicity induced by SCs/MAM-2201 in astrocytes, we applied a novel cellular model, namely three-dimensional (3D) human spheroids that mimic the physiological and biochemical properties of human brain tissues better than the traditional two-dimensional in vitro systems [56,57]. Three-dimensional cultures promote the establishment of complex cell–cell interactions, resembling the in vivo cell–cell and cell–extracellular matrix (ECM) interactions and allowing more innovative in vitro and in silico approaches for studying drug effects [58].

An increasing number of studies have used 3D CNS cultures to investigate the mode and/or mechanism of action of chemicals, even related to events occurring on neurotoxic processes, and to screen chemicals of unknown toxicity [59,60,61,62,63,64,65,66]. Moreover, the use of 3D CNS models derived from human cells provides more human-relevant data compared to animal models, due to inter-species differences.

We aimed to evaluate the impact of different MAM-2201 concentrations on a 3D astrocyte spheroid model in terms of cell stress, growth, apoptosis, death, markers of a reactive state (i.e., glial fibrillary acidic protein, GFAP), cell morphology and density, weight, and diameter. Furthermore, since cell adhesion and the extracellular matrix (ECM) are fundamental to the normal structure and function of three-dimensional tissue spheroids, the effect of MAM-2201 on the integrity of cell–cell interactions was assessed through E-cadherin (E-Cad), a transmembrane glycoprotein adhesion molecule that is necessary to form spheroids [67], and fibrous collagen, one of the major structural components of the ECM. We also evaluated the effect of MAM-2201 on the expression of the CB1 and CB2 cannabinoid receptors.

## 2. Results

### 2.1. Effects of MAM-2201 Exposure on Astrocyte Spheroid Morphology

#### 2.1.1. Spheroid Characterization by Phase-Contrast Microscopy Imaging and Physical Cytometry

**Figure 1:**
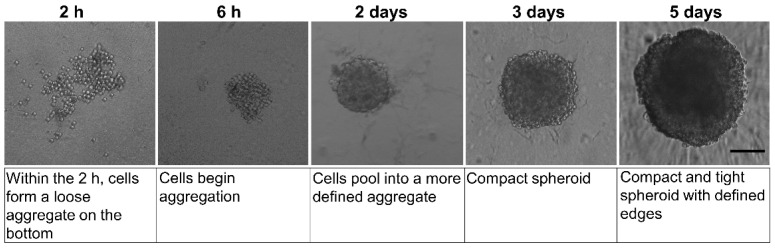
Morphology analysis by phase-contrast microscopy of the D384 spheroid formation over a 5-day period in 96-well spheroid microplate. At 2–6 h after seeding, D384 cells appeared without total compactness. On day 5, complete D384 spheroid formation was observed and this time point was chosen as the starting point for drug treatments. Scale bar: 100 µm.

In this study, we used ultra-low-attachment (ULA) plates to generate 3D spheroids from astrocytes (D384 cell line). D384 cells spontaneously formed 3D spheroids in each well 5 days after plating (200 cells/well). In particular, 2–6 h after seeding into non-adhesion 96-well round-bottomed plates, the D384 cells were aggregated into loose clusters and allowed to settle down to the plate’s bottom (Figure 1).

The D384 spheroid formation typically occurred by the 2nd day (at this point, the cells were aggregated) and the spheroids became tight, compact, and rounder by the 5th day (Figure 1). The spherical shape was maintained throughout the culture process until day 10 [68].

Day 5 (after seeding), when the diameter of the spheroids measured 352.11 ± 2.42 µm, was selected for starting the treatments with the drug at different concentrations (1–30 μM) and then evaluating the effects after 24 and 48 h of treatment (i.e., at day 6 and 7).

**Figure 2:**
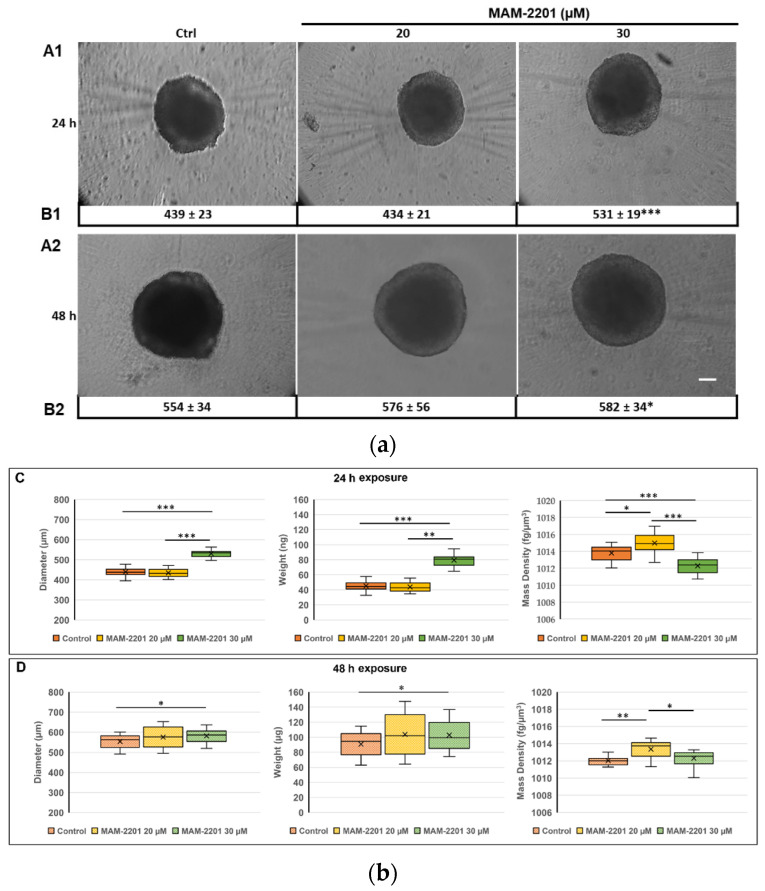
(**a**) Morphology analysis by phase-contrast microscopy of the D384 spheroids after MAM-2201 exposure: MAM-2201 treatments did not cause morphological alterations to the spheroidal structure of spheroids, but induced an increase in the spheroid size at the highest concentration tested (30 µM) for both the 24 h (**A1**,**B1**) and 48 h (**A2**,**B2**) time points considered. Scale bar: 100 µm. (**b**) Biophysical characterization of D384 spheroids after 24 h (**C**) and 48 h (**D**) of MAM-2201 exposure in terms of (left panels) size, (central panels) weight, and (right panels) mass density. Box-and-whisker plots are indicative of the population distribution. * *p* < 0.05, ** *p* < 0.01, *** *p* < 0.001.

The MAM-2201 treatments did not cause morphological alterations to the structure of spheroids, as observed during evaluation by phase-contrast microscopy (Figure 2(A1,A2)).

With regards to the biophysical characterization, the lowest MAM-2201 concentration tested (i.e., 20 µM) did not alter the size or weight of spheroids compared to the control samples, both at 24 and 48 h (Figure 2C,D, left and central panels). On the contrary, the mass density significantly increased at both time points tested (Figure 2C,D, right panel). A different behavior occurred at the highest MAM-2201 concentration tested (i.e., 30 µM), as the size and, consequently, the weight of the D384 spheroids were already significantly enhanced compared to the control after 24 h of treatment (Figure 2C, left and central panels), remaining stable after 48 h (Figure 2D, left and central panels). The effect of 30 µM of MAM-2201 on the mass density reached a maximum at 24 h (Figure 2D, right panel) with a significant decrease compared to the control, while at 48 h, no significant difference was observed (Figure 2D, right panel). Overall, the effects of MAM-2201 on the biophysical parameters were time- and concentration-dependent.

#### 2.1.2. Basic Tissue Structure of the Astrocyte Spheroids by H&E Light Microscopy, ECM, and E-Cadherin Stains

**Figure 3:**
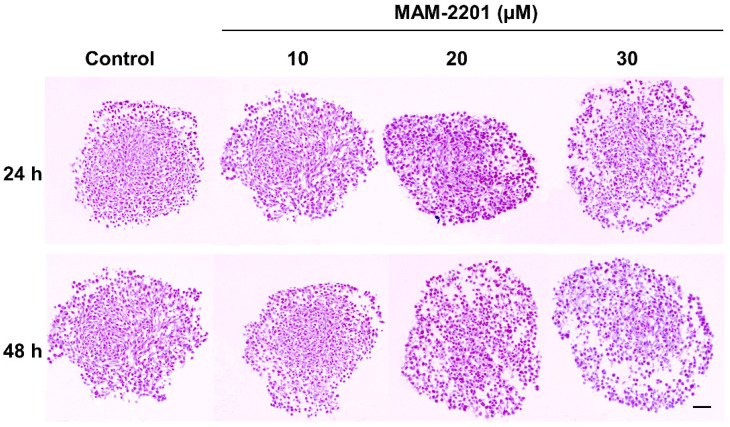
Spheroid histology. The cells were arranged close together and appeared densely packed at a starting density of 200 cells/well in the control spheroid samples at both 24 and 48 h. No necrotic core presence was observed in either the control or treated spheroids. In MAM-2201-treated spheroids, a loose cell packing and poor internal cohesion of cell-to-cell contacts were observed with an evident presence of interstitial spaces between individual cells. Scale bar: 100 μm.

*H&E light microscopy*: Spheroid sections were stained with H&E, revealing the distribution of mass inside the spheroids (Figure 3, Supplementary Figure S1).

In control-matching samples, namely spheroids on day 6 (i.e., 24 h) and 7 (i.e., 48 h), the layers of cells were arranged close together and appeared densely packed with an overall condensed spherical shape (Figure 3, Supplementary Figure S1). The cells were evenly distributed from the inside to the outside of the spheroids and a non-necrotic core was evidenced.

When the spheroids were treated with MAM-2201 at different concentrations and exposure times, loose cell packing and poor internal cohesion in cell-to-cell contacts with the evident presence of interstitial spaces between individual cells were observed starting from 20 µM after 24 h. Similar features were also observed after 48 h of exposure, although the MAM-2201 effect on the D384 cells started at a lower concentration, i.e., from the 10 µM concentration, and became markedly evident at the highest concentrations tested (20–30 µM). No necrotic core or hypoxia was present after the MAM-2201 exposure (Figure 3, Supplementary Figure S1).

An apparent reduction in the cells (spheroids) was observed and the spheroids generally became less spherical, as indicated by the increased diameter (Figure 2b). On the other hand, no necrotic core or hypoxia was present, even after the MAM-2201 treatment.

**Figure 4:**
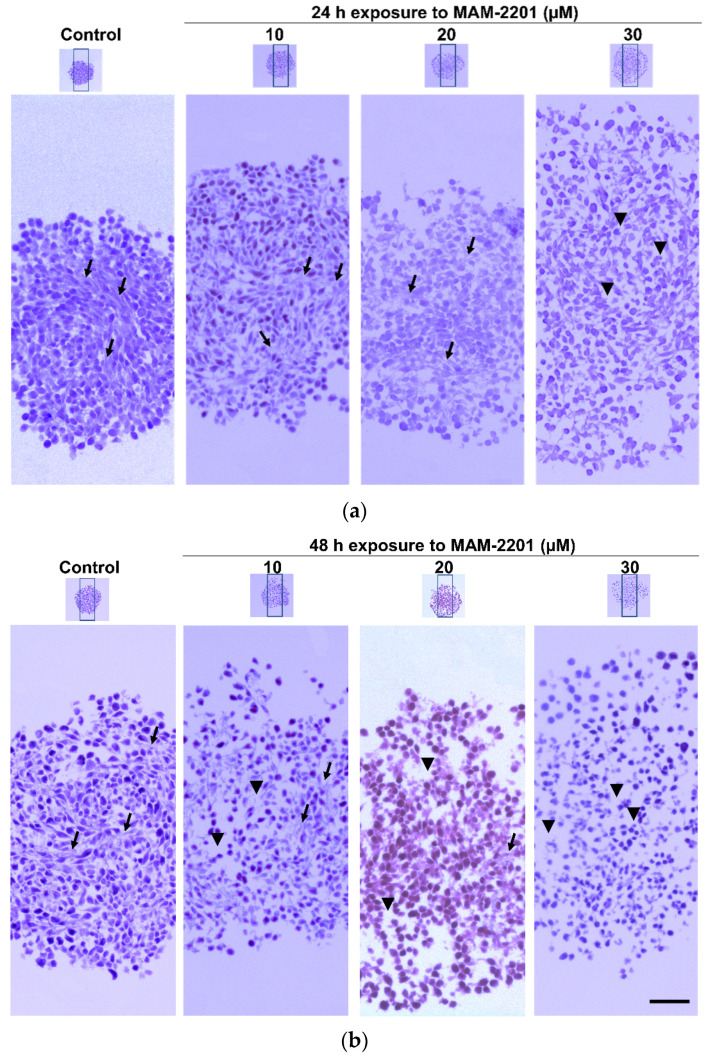
Masson’s trichrome staining. Representative images of trichrome staining in D384 spheroid section treated with or without MAM-2201 after 24 h (**a**) and 48 h (**b**). The rectangle indicates the magnification (10×), black arrows indicate the ECM, and the arrow head indicates the empty spaces (reduction in the amount of collagen fibers). Scale bar: 100 μm.

*ECM and E-cadherin evaluation*: Masson’s trichrome staining showed a well-structured organization of the ECM in the control spheroid section on days 6 (i.e., 24 h) and 7 (i.e., 48 h) (Figure 4, Supplementary Figure S2).

A progressive ECM disassemble started at ≥20 µM of MAM-2201 after 24 h: the ECM appeared less compact with more empty spaces compared to the control, as visualized by a reduction in the amount of blue stain (collagen fibers) throughout the section of spheroids (Figure 4a, Supplementary Figure S2a). The effects were exacerbated after 48 h of treatment: a progressive ECM staining decrease was observed starting at ≥10 µM of MAM-2201 (Figure 4b, Supplementary Figure S2b). These results were consistent with those showed by H&E staining (Figure 3, Supplementary Figure S1).

Decreased levels (about 30%) of E-Cad at 20 and 30 µM after 48 h were also observed (Table 1). These results, together with those showed by the H&E and ECM stains (Figure 3 and Figure 4, respectively), indicated that the treated D384 spheroids were composed of some cells without any obvious close cell–cell adhesion, showing a looser aggregation phenotype compared with the control spheroids.

### 2.2. Cell Viability Evaluation

**Figure 5:**
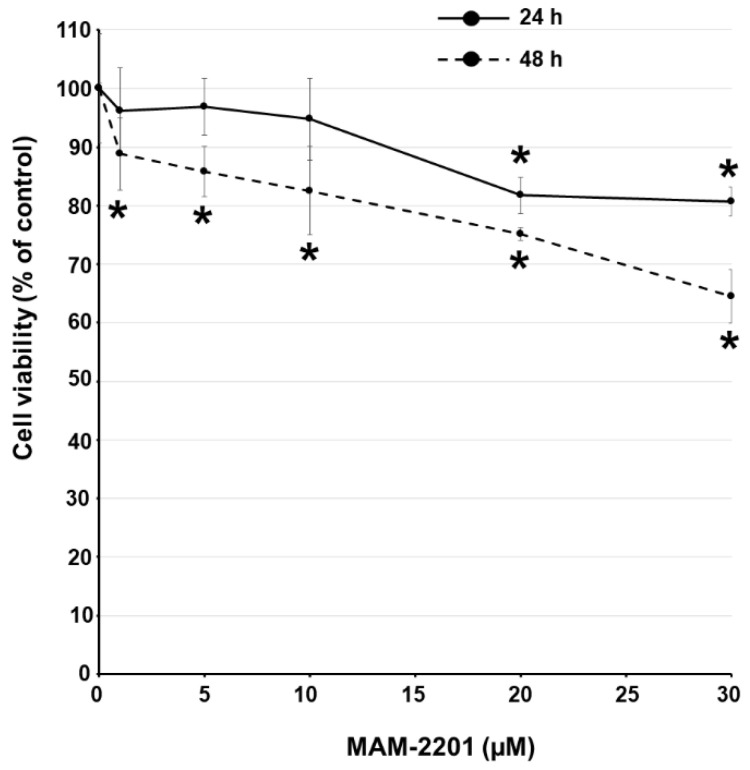
Cell viability evaluation by trypan blue (TB) exclusion test; cytotoxicity effects after 24 and 48 h of exposure to increasing concentrations of MAM-2201 (1–30 μM). Data are normalized to the mean value obtained under control conditions, expressed as percentages (% of control), and plotted as the means ± S.E. * *p* < 0.05, statistical analysis by one-way ANOVA followed by Tukey’s multiple comparisons test.

A cell viability evaluation by TB indicated a decrease in viable cells (~20%) at the higher MAM-2201 concentrations tested (20–30 µM) after 24 h. The effects on cell mortality were more evident after 48 h of treatment: the cell decrease (~10–15%) appeared at the lower concentrations (from 1 to 10 µM) with a further cell survival reduction (25–35%) at higher concentrations (20–30 µM) (Figure 5).

Since the viability analysis, evaluated by a TB test, changed over time (the test sample should be counted in bright fields within 5 min after mixing with trypan blue) and the viability of a cell population may be underestimated [69], an additional sensitive cell viability measurement over time in a live-cell format was performed by using a nonlytic, bioluminescent method. This test allowed cell viability to be measured in real time by analyzing the metabolically active cells after MAM-2201 exposure over time, i.e., from 1 up to 48 h.

**Figure 6:**
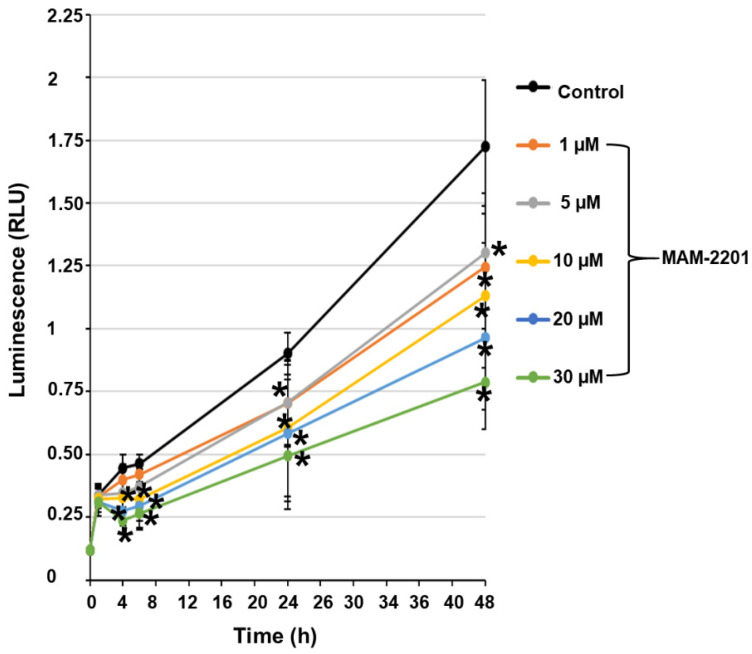
Cell viability over time. Time- and concentration-dependent MAM-2201 effects: the effect started at ≥10 µM of MAM-2201 after 4 h and at ≥5 µM after 24 h of exposure. Results are provided as means ± S.E. of two independent experiments performed in eight replicates. * *p* < 0.05, statistical analysis by one-way ANOVA followed by Tukey’s multiple comparisons test.

A significant concentration-dependent inhibition of cell proliferation was observed from 5 to 30 µM and over time in astrocytes. The effect was already significant after 4 h of exposure to MAM-2201 at ≥10 μM, and at ≥5 μM after 24 h (Figure 6).

### 2.3. Evaluation of Caspase-3/7 Activity in Astrocyte Spheroids after MAM-2201 Exposure

**Figure 7:**
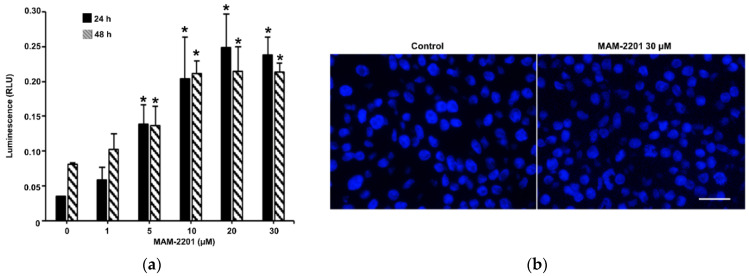
(**a**) Composite images showing the evaluation of caspase-3/7 activity and (**b**) apoptotic cells in D384 spheroid sections after MAM-2201 exposure. The caspase-3/7 activity was impaired: an increase in the activity levels was observed in D384 spheroids starting from 5 to 30 μM for both time points considered (24–48 h). Apoptotic cells detected using Hoechst 33,258 staining were not visible after 48 h of exposure to MAM-2201 at the highest concentration tested (30 μM). The data obtained are provided as the means of the luminescence values (RLU) ± S.E. * *p* < 0.05, statistical analysis by one-way ANOVA followed by Tukey’s multiple comparisons test. Representative images in fluorescence microscope were taken using a magnification of 40×; scale bar: 50 μm.

The caspase-3/7 activity was assessed to determine if apoptosis was involved in the mechanism of cytotoxicity induced by MAM-2201 in astrocyte spheroids, and it was evaluated after 24 and 48 h of exposure to increasing concentrations of MAM-2201 (1–30 μM). An increase in the caspase-3/7 activity was detected (4–7-fold from 5 to 30 μM) after 24 and 48 h of exposure (Figure 7a). However, no morphological hallmarks of apoptosis in the treated cells were observed with immunofluorescence (Figure 7b).

### 2.4. Evaluation of CB1 and CB2 Receptors

**Figure 8:**
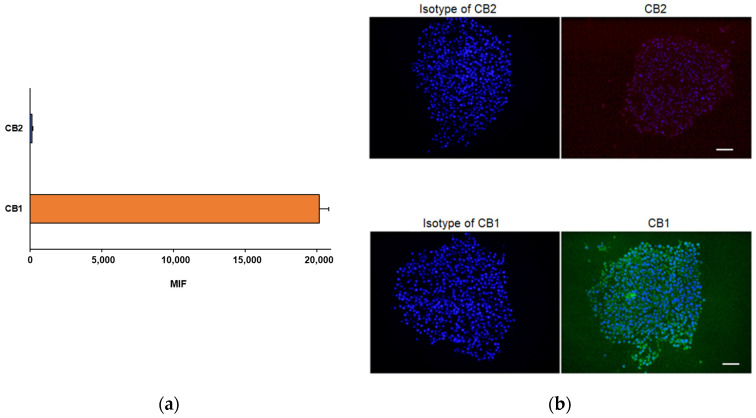
(**a**) Flow cytometric and (**b**) immunofluorescence analyses of the cannabinoid receptor expression in D384 spheroid sections. A higher expression of CB1 receptors was observed compared to CB2 expression, as demonstrated by both flow cytometry and immunofluorescence staining. The flow cytometry data are expressed as median fluorescence intensities (MFIs) and represent the means ± S.D. The images show representative fluorescence-merged microphotographs with CB1-positive (green fluorescence) and CB2-positive (red fluorescence) areas in D384 spheroid sections. Nuclei were stained with Hoechst 33258. Scale bar: 100 μm.

The three-dimensional astrocyte cultures, evaluated by flow cytometry, expressed CB1 receptors, while a weak signal was detected for CB2 receptors (Figure 8a).

The evaluation of CB1 and CB2 receptors by immunofluorescence confirmed, as evidenced by the flow cytometry, the abundant expression of CB1 receptors (green-labelled) in astrocytes as well as very weak immunoreactivity for CB2 receptors (red signal) (Figure 8b).

**Figure 9:**
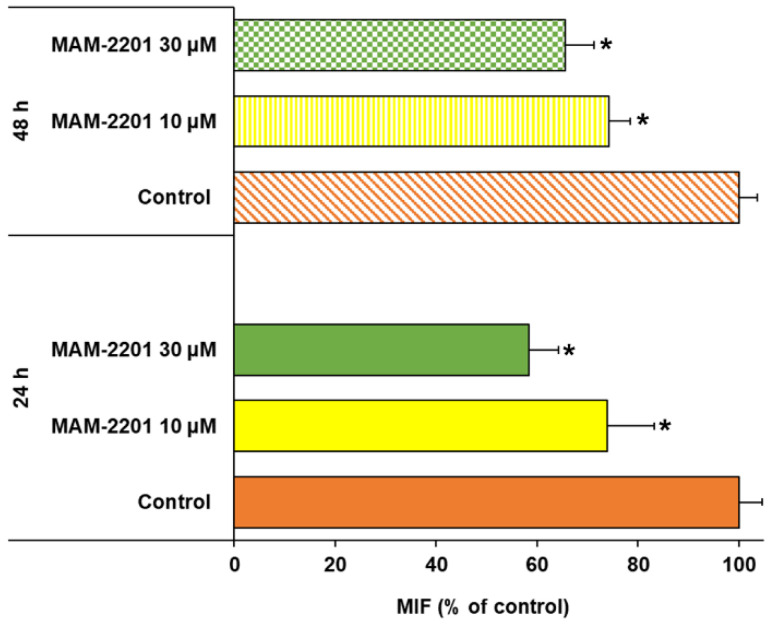
Flow cytometry analysis of cannabinoid receptor expression in D384 spheroids after 24 and 48 h of exposure to MAM-2201 (10 and 30 µM). Data are expressed as MFI percentages (% of respective control) and plotted as the means ± S.D. * *p* < 0.05, statistical analysis by one-way ANOVA followed by Tukey’s multiple comparisons test.

When the spheroids were treated with MAM-2201, the CB1 receptor expression was reduced by about 25% at the lower MAM-2201 concentration tested (10 µM) after 24 h of exposure, with exacerbation at the highest concentration (30 µM) (about 35% reduction). The effect of MAM-2201 on the CB1 receptor expression persisted for up to 48 h (Figure 9).

**Figure 10:**
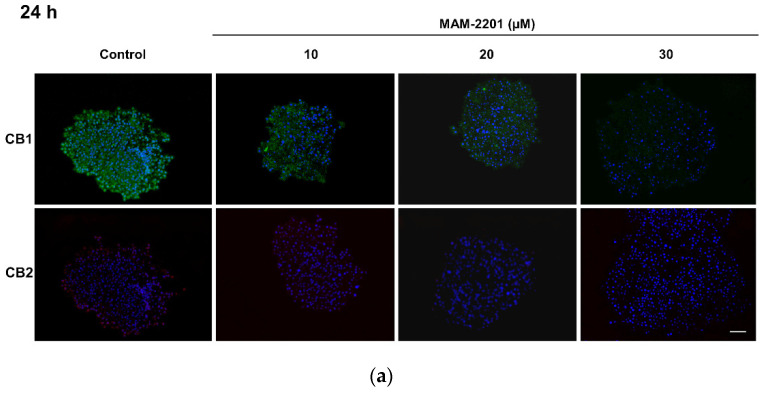

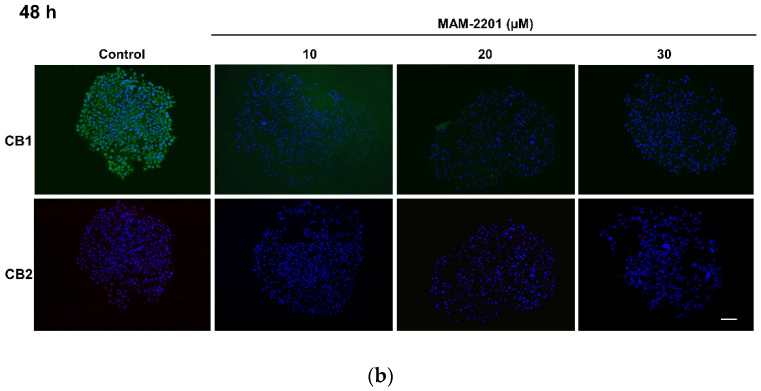
Immunofluorescence analysis of the cannabinoid receptor expression in astrocyte spheroid section after (**a**) 24 and (**b**) 48 h of exposure to MAM-2201. A loss of the fluorescence signal of CB1 (green fluorescence) was observed in D384 spheroid sections, starting at 20 µM after 24 h and starting at 10 µM after 48 h. CB2 showed very low levels of the fluorescence intensity (red) in both the D384 spheroid control and treated spheroids after 24 h, which disappeared after 48 h in treated D384 spheroids. Nuclei were stained with Hoechst 33258. Scale bar: 100 μm.

The evaluation of the receptors by immunofluorescence also showed a reduction in the CB1 receptor green fluorescent signal in a concentration-dependent manner, starting from 20 µM after 24 h and from 10 µM after 48 h (Figure 10a). The weak red signal of CB2 (evidenced in the control) completely disappeared after exposure to the low MAM-2201 concentration (Figure 10b).

### 2.5. Effects on GFAP

**Figure 11:**
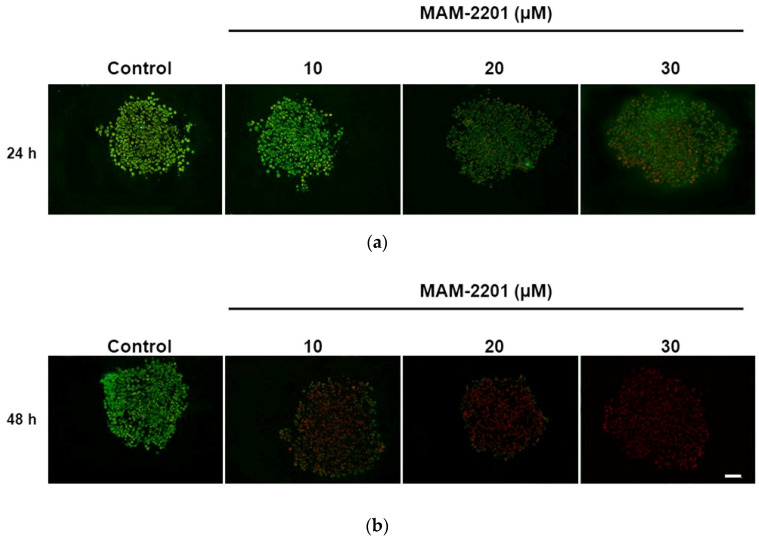
Immunofluorescence analysis of GFAP in astrocyte spheroid sections after (**a**) 24 and (**b**) 48 h of exposure to MAM-2201 (10, 20, and 30 μM). A loss of the GFAP fluorescence signal (green) was observed in D384 spheroids, starting at 20 μM after 24 h, and was exacerbated and worsened (starting at 10 μM) after 48 h of exposure. Nuclei were stained with propidium iodide. Scale bar: 100 μm.

The astrocyte cultures abundantly expressed the GFAP (green color) (Figure 11), and when the spheroids were treated with MAM-2201, the expression diminished in a concentration-dependent manner after 48 h. A loss of the fluorescence intensity of the GFAP was observed in the D384 spheroids, starting at 20 μM after 24 h (Figure 11a). The effect was exacerbated and also worsened (starting at 10 μM) following 48 h of exposure (Figure 11b).

## 3. Discussion

The present in vitro findings demonstrate the cytotoxicity of MAM-2201 on human astrocyte spheroids (D384), and support the use of these human 3D cell-based models as species-specific in vitro tools suitable for the evaluation of neurotoxicity induced by other SCs.

Considering the consequences on human health due to the use of these new psychoactive substances and their spreading on the market, the application of a cellular model for neurotoxicology research (applying human-derived CNS cells in three-dimensional cultures) may provide a means for understanding the neurotoxicity of these substances. We utilized a human 3D astrocyte in vitro culture generated from the D384 cell line (D384) cultured using an ultra-low-attachment (ULA) surface enabling 3D spheroid formation in 96-well round-bottomed plates. A ULA-3D culture is more widely used, since it is compatible with many cell lines, initiates by self-assembly, and generates a complex, tissue-like ECM in addition to its simplicity and inexpensive nature [70,71].

Our investigation demonstrated that the D384 cells form compact spheroids: once the astrocytes were seeded in the ULA plate, the cells aggregated and formed a spherical shape within few days. The layers of astrocytes in spheroids were arranged close together and appeared densely packed with an overall condensed spherical shape. The cells were evenly distributed from the inside to the outside of the spheroids without evidence of necrotic core. The value of circularity increased over a few days (~7 days), indicating that the cells in the ULA plate easily self-organized and formed a circular spheroid.

The cell lines that formed compact spheroids typically possessed high levels of extracellular content (ECM) and E-cadherin, which play an important role in cell adhesion [72]: these features were observed in the present astrocyte spheroids (control, not treated), with the spheroids showing abundant ECM content and E-cadherin. Typically, the initial aggregation of cells is initiated by the integrin-mediated attachment to ECM molecules, and the cells are aggregated compactly by E-cadherin mediation [73]. E-cadherin’s adhesive function at the cell surface allows the cells to hold together, facilitates other cell–cell interactions, and physically blocks the movement of cells [74].

In the formed astrocyte spheroids, we also demonstrated the expression of CB1 receptors and the gold standard marker of the astrocyte GFAP, an intermediate filament protein present in the astrocyte cytoskeleton.

The MAM-2201 treatment had a clear cytotoxic effect on the present 3D astrocytes, as evidenced by the significant decrease in cell proliferation over time and the increase in dead cells. The inhibition of cell proliferation due to MAM-2201 exposure was concentration- and time-dependent. Cell mortality (~20%) was detected at the higher concentrations tested (20–30 µM) after 24 h, but it was more evident after 48 h of treatment in that the cell decrease (~10–15%) occurred at the lower concentrations (from 1 to 10 µM) with mortality exacerbation (25–35%) at higher concentrations (20–30 µM).

Moreover, by using a three-dimensional model that allowed us to analyze some aspects of cell behavior (e.g., growth, cell adhesion, etc.) in a more physiological setting than a 2D cell culture, it was possible to highlight modifications to the spheroid size after the MAM-2201 treatment as well as changes to the basic tissue spheroid structure. The astrocyte spheroid size was apparently enhanced. This may have been due to the dispersion of the remaining live cells in association with a decreased amount of ECM and E-cadherin. It might have been that as the MAM-2201 treatment progressed, the cell-to-cell and cell-to-matrix interactions were disrupted, thus leading to cell disaggregation. As the cells at the edges fell apart, the shape of the sphere collapsed.

Furthermore, in these 3D astrocytes, we observed a decrease in CB1 receptors after the MAM-2201 treatment (at ≥10 µM for 24 and 48 h), which was associated with a decrease in the E-cadherin and GFAP, and an increase in caspase-3/7 activity.

It is important to consider the role that astrocyte-induced plasticity may be playing in the behavioral sequelae of insults, such as stressors or neurotransmitters (endogenous or exogenous—e.g., synthetic drugs), which, through their receptors located in astrocytes, directly induce intracellular cascades. The latter ultimately introduces changes in the morphology/physiology of astrocytes that alters the normal functioning of tripartite synapses in a pathophysiological direction that is known to drive behavioral sequelae [53,55].

Recently, it has been hypothesized that there is an interaction between the CB pathway and the IGF-1R/AKT pathway, which are known to regulate several physiological cellular processes, including cell proliferation, survival, growth, differentiation, and metabolism [75]. The same authors also suggest a relation between CB1, CB2, and E-cadherin expression through IGF-1R/AKT/GSK-3β axis regulation. Indeed, a change in the E-cadherin levels is one of the hallmarks of an epithelial–mesenchymal transition, which is a crucial program in the regulation of cell motility and invasion. E-cadherin loss promotes a reduction in cell–substrate adhesion and causes a significant disruption to the normal organization of the microtubule and actin cytoskeletons [76].

The increase in caspase-3/7 activity started from 5 µM after 24 h of treatment and persisted up to 48 h without the association of the morphological hallmarks of apoptosis. Indeed, while the mechanisms controlling the activation of caspases and their targets are well established in the context of apoptosis and inflammation, accumulating recent evidence also supports a non-apoptotic and non-inflammatory function of caspases [77,78,79]. The non-proteolytic functions of caspases have, in particular, been evidenced in the regulation of cell survival, proliferation, and differentiation [80,81,82]. Besides, over the last decade, evidence has been gathered detailing non-apoptotic roles for caspases in astrocytes, neurons, oligodendrocytes, and microglia [83,84,85,86,87,88]. In particular, in the CNS, caspase-3 participates in cytoskeletal remodeling in neurons and astrocytes [83,89], and in the differentiation of astrocyte subpopulations [90]. Notably, a caspase-3-cleaved GFAP in cortical astrocytes was detected following excitotoxic injury [83]. Recent evidence suggests that caspase activation in astrocytes may be required for the full expression of the reactive phenotype [91]. It might be that high levels of caspase activation lead to caspase-dependent apoptosis, while limited caspase activation may reveal mainly the nonapoptotic functions (proliferation, differentiation, intercellular communication through cytokine release, and NF-kB activation) [78]. Moderate activation could be the situation observed in MAM-2201-treated astrocyte spheroids, where a mild increase in caspase-3/7 without apoptosis and a decrease in the GFAP occurred. Recent studies have demonstrated that THC exposure for 72 h causes significant modifications to astrocyte morphology, possibly reflecting functional alterations, as evidenced by the decrease in GFAP expression and the morphological alterations of astrocyte branches [43]. In both the CA1 stratum pyramidalis and stratum radiatum of THC-treated hippocampal slice cultures, the astrocytes showed marked signs of clasmatodendrosis, an irreversible astrocytic degeneration characterized by the dissolution of their branches [92]. It is therefore plausible that astrocyte clasmatodendrosis caused by continuous exposure to THC, which leads to the spatial disorientation of astrocytes, and the disruption of the astrocytic syncytium may decrease the maintenance of healthy synapses and synaptic connectivity, and this, in an in vivo situation, may also play a role in decreasing neuronal homeostasis.

The common paradigm for identifying THC-like effects, the cannabinoid tetrad, suggests that many synthetic cannabinoid effects are mediated primarily by the CB1 receptor. This receptor undergoes downregulation and desensitization through repeated stimuli, but can be recovered within days to weeks of cessation [93]. Markedly, synthetic cannabinoids are more potent full CB1 agonists compared to THC, which is a partial CB1 receptor agonist.

Notably, we observed a decrease in CB1 receptors in the MAM-2201-treated astrocytes. The involvement of astrocytes in THC-dependent memory deficits has been recently demonstrated [94]. Moreover, Cong et al. [95] found that astroglial CB1 receptors mediate aversive effects of the synthetic CB CP 55,940 (a member of the cyclohexyl phenols and a synthetic analog of Δ9-THC, the major psychoactive component of marijuana).

In astrocytes, the activation of CB1Rs can modulate the astroglial metabolism [96], mediate neuron–astrocyte communication [47], and cause synaptic and memory impairments [97].

Indeed, although neurons highly express CBR1, the role of CBR1 in astrocytes is being increasingly appreciated and recognized for its contribution to the detrimental behavioral effects associated with cannabis [42,98,99,100,101]. In particular, recent studies have shown that the detrimental effects of Δ9-THC on learning and memory in mice are mediated by the astrocyte CBR1 [45,94,97,102,103], the activation of NF-κB signaling, and the up-regulation of cyclooxygenase-2 (COX-2), which might lead to excessive glutamate release by astrocytes. The latter is responsible for glutamate excitotoxicity, a mechanism known to be involved in many types of neurodegenerative diseases. On the other hand, excitotoxic injury may cause the cleavage of the GFAP and then clasmatodendrosis [43]. It may also be that astrocytes, in an attempt to regulate the interstitial concentration of glutamate (and likely, other ions) [104], can become so acidotic that they are lethally injured and undergo clasmatodendrosis [92,105]. Changes in the GFAP probably not only reflect a structural consequence, but also a functional consequence for the astrocyte physiology, since this protein has been implicated in cell-to-cell communication, the anchoring of proteins, and the reaction to brain insults [106].

Altogether, the astrocyte effects observed after the MAM-2201 treatment in our experimental study can be mediated in part by CB1 receptors, in part by the permeation of the cell membrane due to its lipophilicity, and possibly through other types of receptors, e.g., vanilloid receptors, as demonstrated for other SCs in C6 rat gliomas [107].

In summary, the present in vitro findings indicate that MAM-2201 causes adverse effects on astrocyte spheroids of human origin (D384), affecting the cell grow and viability, size and structure, CB1 receptors, GFAP, E-cadherin and ECM, and caspase-3/7 activity.

Regarding the cytotoxic effects observed on the 3D spheroid model of astrocytes, they appeared more marked than those observed in the same human astrocyte line cultivated in a monolayer (2D), as we previously reported [31]. Moreover, the spheroid model allowed for the detection of the inhibition of cell growth proliferation caused by MAM-2201, starting at low concentrations (5–10 µM), early (after 4 h), and lasting up to 48 h, as well as the modifications to the spheroid size and changes to the basic tissue structure.

## 4. Materials and Methods

### 4.1. Culture Reagents

Dulbecco’s modified Eagle medium (DMEM), all cell culture reagents, and fetal bovine serum (FBS) were purchased from CliniSciences (Guidogna Montecelio, Italy). A 75 cm^2^ tissue culture flask with vented filter caps (Corning, Schnelldorf, Bavaria, Germany) and ULA 96-well round-bottomed plates (Corning, Schnelldorf, Bavaria, Germany) were acquired from Merck Life Science S.r.l. (Milan, Italy).

### 4.2. Drug and Reagents

[1-(5-fluoropentyl)-1Hindol-3-yl](4-methyl-1-naphthalenyl)-methanone (MAM-2201) (Cayman) was purchased from LGC Standards S.r.l. (Milan, Italy) (Authorization of the Italian Ministry of Health—General Directorate for Medical Devices and Pharmaceutical Service for the purchase, possession and use of analytical standards of psychotropic substances “DPR 309/90 art.60, DM 15.02.1996 and DM 03.08.2001” issued to the pharmacy unit of our ICS Maugeri Hospital—Pavia for the management of psychotropic substances in the clinical and laboratory setting).

Harris hematoxylin, the eosin Y-solution (0.5%), the alcoholic solution, and the bio clear solution were purchased from Bio-Optica Milan Spa (Milan, Italy). The trypan blue solution (0.4%; Corning, Manassas, VA, USA) was obtained from VWR (Milan, Italy). The RealTime-Glo™ MT Cell Viability and Caspase-Glo^®^ 3/7 assays were acquired from Promega (Milan, Italy). The GFAP antibody (mouse IgG2b kappa light chain) (Santa Cruz Biotechnology, Dallas, TX, USA) was purchased from D.B.A. Italia S.r.l (Segrate, Italy). Primary antibodies (mouse IgG1 kappa light chain) conjugated to Alexa-Fluor^®^ 488 for CB1 (Santa Cruz Biotechnology, Dallas, TX, USA), primary antibodies (rabbit IgG polyclonal) conjugated to Alexa-Fluor^®^ 594 for CB2 (Bioss, Boston, MA, USA), and the secondary antibody (mouse IgG2b kappa light chain) conjugated to Alexa-Fluor^®^ 488 (Cohesion Biosciences, London, UK) for the GFAP were acquired from D.B.A. Italia S.r.l (Segrate, Italy) and CliniSciences (Guidogna Montecelio, Italy), respectively. The mouse IgG1 kappa isotype control conjugated to Alexa-Fluor^®^ 488 (Invitrogen, Waltham, MA, USA) for CB1 and the rabbit IgG isotype control conjugated to Alexa-Fluor^®^ 594 (Bioss, Boston, MA, USA) for CB2 were obtained from Life Technologies Italia (Monza, Italy) and CliniSciences (Guidogna Montecelio, Italy), respectively. Hoechst 33258 (Invitrogen, Waltham, MA, USA) was purchased from Life Technologies Italia (Monza, Italy). The trichrome stain (Masson) kit (Sigma-Aldrich, Schnelldorf, Bavaria, Germany), and propidium iodide (Sigma-Aldrich, Schnelldorf, Bavaria, Germany) were acquired from Merck Life Science S.r.l. (Milan, Italy). The Human E-Cad (E-Cadherin) ELISA Kit (Elabscience, Houston, TX, USA) was acquired from Microtech (Naples, Italy).

### 4.3. Astrocyte Spheroid Model

Spheroid cultures were obtained from the human astrocyte D384 clonal cell line, an established line derived from a human astrocytoma [108], as previously described by De Simone et al. [68]. Briefly, D384 cells were cultured in a monolayer in DMEM medium supplemented with 10% heat-inactivated FBS, 2 mM L-glutamine, 50 IU/mL penicillin, 50 μg/mL streptomycin, and 1% sodium pyruvate. The D384 cells were trypsinized when about 80% confluence was reached and re-seeded, based on the D384 proliferative capacity and their cell cycle time (about 9 h), at a cell density of 200 cells/200 µL/well in ULA 96-well round-bottomed plates to obtain spheroids with a diameter of 352.11 ± 2.42 µm on day 5 [68]. Spheroid sizes between 100 and 500 μm have been commonly accepted to be representative of healthy spheroid structures, owing to sufficient oxygen and nutrition transport [109]. Specifically, small spheroids (<100 μm) might fail to display the complexity of real tissues with low growth rates, whereas large spheroids (>500 μm) might have pronounced necrotic cores due to the diffusion limitations for oxygen and nutrition.

### 4.4. Treatment with MAM-2201

The spheroids were cultured and monitored for 5 days: old medium was carefully changed with fresh medium, taking care not to disturb the spheroids.

On day 5, the spheroids were treated (single dose) with different MAM-2201 concentrations (1, 5, 10, 20, or 30 µM) and evaluated after 24 and 48 h (corresponding to spheroids on days 6 and 7 after seeding). The MAM-2201 solutions were prepared by dissolving the powder in DMSO and then diluting in complete DMEM medium. Fresh solutions were prepared immediately before using, and then the spheroids were incubated under normothermic (37 °C) conditions in a humidified atmosphere (95% air/5% CO_2_). Control samples were treated with DMSO, which was used to dissolve the MAM-2201 powder (1 mg). In particular, the control samples were treated with 0.5% DMSO, with the concentration corresponding to the higher DMSO concentration used for the higher MAM-2201 concentration (30 μM) tested. The final concentration of DMSO (0.5%, *v*/*v*) in each well was demonstrated to be compatible with cell viability.

The tested MAM-2201 concentrations (1–30 μM) were selected based on literature findings from in vitro studies carried out on brain cultures (rodent) that evidenced early cytotoxic effects (2–24 h, from 10 to 30 μM MAM-2201 treatment): MAM-2201 acted as a CB1R agonist and induced acute cytotoxicity by the involvement of caspase cascade-mediated apoptosis [24,25,27,28,29]. Moreover, we recently demonstrated MAM-2201 cytotoxicity in a human astrocyte line cultured in a monolayer (2D), in which the density decrease was noticeable at 30 μM after 24 h and at 10 μM after 48, and the caspase-3/7 activity impairment was evidenced at 5–30 μM, although transitorily (i.e., after 3 h of exposure only) [31].

### 4.5. Cell Morphology Analysis by Phase-Contrast Microscopy

Astrocyte spheroids were observed under inverted microscopy in the bright field mode (equipped with a 10× objective) after MAM-2201 (1–30 µM) exposure to evaluate the healthy status of the cells, the spheroid growth/size, and the morphological changes. Digital photographs from each well/spheroid were captured after 24 and 48 h of treatment with a camera (Canon powershot G8) and stored on a PC. In order to analyze the spheroid size, a calibration slide was used, and then the pictures were processed using Image J software 1.51 (NIH, Bethesda, MD, USA). The color-captured images (n = 6), derived from each condition, were converted to binary images and analyzed with the ‘‘measure tool”. The data analyses were performed in Microsoft Excel.

### 4.6. Frozen Sections of Astrocyte Spheroids for Histochemistry and Immunofluorescence Analyses

After each exposure time point, the culture medium was carefully removed from each well and at least 25–30 spheroids per condition were pooled in a microcentrifuge tube, washed with phosphate-buffered saline (1 mL/tube PBS), fixed in a 4% paraformaldehyde solution (PF; for 60 min at room temperature (r.t.)), and re-washed. Then, the spheroids were cryoprotected. Specifically: the spheroids were submerged in 10% sucrose in PBS solution for 30 min at r.t., then centrifuged, re-submerged in a 20% sucrose solution for 30 min at r.t., and finally, submerged in a 30% sucrose solution overnight at +4 °C. The next day, the spheroids were centrifuged and embedded in an optimal cutting temperature compound (OCT). Five μm-thick cryostatic sections were cut by using a cryostat (Leica CM 1950, Leica Microsystems, GmbH, Wetlzar, Germany) and deposed on silane prep slides for the subsequent staining and labelling processes or stored at −80 °C.

The hematoxylin and eosin stain and Masson’s trichrome stain were used to assess the basic tissue structure of the astrocyte spheroids.

#### 4.6.1. Hematoxylin and Eosin (H&E) Staining

The spheroid sections were processed using histology-staining instruments (Leica ST5020, Leica Microsystems, GmbH, Wetlzar, Germany). Firstly, the spheroid sections were allowed to air dry (2 min at r.t.), then rinsed with tap water (2 min) and stained with Harris hematoxylin (3 min), and again rinsed with tap water (3 min). Afterwards, the sections were covered with a 0.5% alcoholic eosin Y solution (2 min) and dehydrated in a 95% alcoholic solution (90 s), in a 100% alcoholic solution (30 s, twice), and finally in a bio clear solution (clearing agent) (90 s, twice), and were then mounted with Neo-Mount. The images were acquired using a light microscope (Carl Zeiss AXIOSKOP 40/40FL microscope, Milan, Italy) equipped with an objective (20×) lens and a digital camera (AxioCam MRc5 Carl Zeiss, Milan, Italy).

#### 4.6.2. Masson’s Trichrome Staining

The sections obtained from MAM-2201-treated spheroids were stained with Masson’s trichrome kit by following the producers’ instructions.

The astrocyte spheroid sections were first left to equilibrate at r.t. (10 min), and were then rehydrated in deionized water (10 min at r.t.). The sections were covered with Bouin’s solution (pre-warmed at 56 °C) for 15 min, and were then cooled in tap water and washed (three times; 5 min for each washing) to remove the yellow color from the sections. The slides were stained (5 min) using working Weigert’s iron hematoxylin solution (Part A plus Part B), washed again in tap water (three times; 5 min for each washing), and rinsed using deionized water (three times; 5 min for each washing). Then, they were stained using the Biebrich scarlet-acid fucshin (5 min), and after the washing (three times in deionized water; 5 min for each washing), the sections were stained in a working phosphotungstic/phosphomolybdic acid solution (5 min), then in an aniline blue solution (5 min), and then in a 1% acetic acid solution (2 min). Finally, the sections were rinsed in deionized water (three times; 5 min for each washing), dehydrated through an alcohol scale (80%, 90%, 100%, 100%:xylene), cleared in xylene, and then mounted with Neo-Mount. Images were acquired in the brightfield (20× magnification; Zeiss AXIOSKOP 40/40FL microscope).

### 4.7. E-Cadherin Biochemical Evaluation

E-cadherin was measured using a human E-Cad (E-cadherin) ELISA kit by the sandwich-ELISA technique. Standards (0–10 ng/mL) and samples (control and treated with MAM-2201) derived from cell lysates were collected by following the manufacturer’s instructions and were then pipetted into wells to bind to the antibody specific to human E-Cad into a micro-ELISA plate. Biotinylated detection antibodies targeting human E-Cad and the avidin-horseradish peroxidase (HRP) conjugate were added successively into each well. After incubation, washing for unbound components was performed. When the substrate solution was added, only wells containing human E-Cad, the biotinylated antibody, and the avidin-HRP complex were colored. The enzyme–substrate reaction was terminated by the addition of a stop solution. The optical density (OD) was measured using microspectrophotometry (BioRad, Benchmark, Segrate, Italy) at a wavelength of 450 nm. Each sample was run in duplicate. The OD value was proportional to the human E-Cad concentration. The concentrations of E-Cad were determined by extrapolating the values on a standard curve (0.16–10 ng/mL) and were represented as the % of the control (about 1 ng/mL).

### 4.8. Expression of CB Receptors and GFAP by Immunofluorescence Analysis

The expression of the CB1 and CB2 receptors and the astrocyte marker as the GFAP by immunofluorescence analysis were evaluated after the MAM-2201 treatments by applying the following protocol: after rehydration, the cryo-sections of the D384 spheroids were permeabilized (with 0.25% Triton X-100 in PBS solution for 10 min at r.t.) and incubated for 30 min in a blocking buffer (1% BSA in PBS). Afterward, the spheroids were incubated with primary antibodies conjugated to Alexa-Fluor^®^ 488 (green color) or Alexa-Fluor^®^ 594 (red color) for CB1 and CB2, respectively, used at a dilution of 1:100 in 1% BSA solution for 60 min at r.t. in the dark. Next, the spheroid sections were washed with PBS (three times; 5 min for each washing) and the nuclei were detected using Hoechst 33258 (blue color) (5 µM for 10 min at r.t.), and they were finally mounted with Fluoroshield.

For the GFAP immunostaining, the astrocyte spheroid sections were incubated with primary antibody non-conjugated anti-GFAP (1:100, diluted in 1% BSA solution) overnight at +4 °C. The next day, the primary antibody was removed and the sections were washed (three times with PBS; 5 min for each washing) and stained with secondary antibody conjugated to Alexa-Fluor^®^ 488 (green color) (dilution 1:100) for 60 min at r.t. in the dark. After washing with PBS (three times; 5 min for each washing), the nuclei were detected using propidium iodide (PI) (red color) (1 µg/mL, for 10 min at r.t.), and then the slides were mounted with Fluoroshield.

The fluorescence images were acquired using a CX41 Olympus fluorescence microscope (Olympus, Segrate, Italy), with the excitation light being provided by an EPI LED Cassette and combined with a digital camera. Digital images of the eight randomly selected microscopic fields (for each receptor and GFAP) were captured using a 20X objective lens, and the measurement conditions were the following: 470 nm excitation (T% = 40), 505 nm dichroic beamsplitter, and 510 nm-long pass filter.

### 4.9. Detection of CB Receptor Expression by Flow Cytometry Analysis

The cannabinoid receptor expressions were analyzed in single cells within spheroids using flow cytometry. Briefly, spheroids (n = 18 for each condition) were washed with PBS and dissociated with a trypsin solution (200 µL/well, up to 5 min at 37 °C). Afterwards, an equal volume of medium was added and the cells were resuspended and counted using the Burker chamber to determine the cell viability by a trypan blue exclusion test. The cells were fixed, permeabilized, and incubated with primary antibodies conjugated to Alexa-Fluor^®^488 or 594 against CB1 (1 µg/tube) and CB2 (1 µg/tube) or isotype-matched Abs (negative controls) using the BD cytofix/cytoperm kit according to the manufacturer’s instructions. The isotype-matched Abs directed against irrelevant antigens, employed to assess background staining and specificity, demonstrated no background staining due to non-specific antibody binding.

Then, the cells were washed (0.5% BSA solution) and the samples were analyzed using a two-laser flow cytometer (FACSCantoII), and managed with Diva Software (BD Biosciences). The values were expressed as MFIs (median fluorescent intensities).

The antibodies applied to reveal the CB1 and CB2 receptors were able to detect the amino acids 1–150 of CB1 and 251–360 of CB2 of human origin.

### 4.10. Real-Time-Glo MT Cell Viability Assay

To monitor the cell responses to MAM-2201, the cell viability was evaluated in astrocyte spheroids over time (1, 4, 6, 24, and 48 h) after the MAM-2201 treatments (1–30 μM), according to the protocol supplied by the manufacturer.

The cell viability measurement was performed in real time: the luminescence was read from 1 to 48 h by a Fluoroskan microplate fluorometer (Thermo Scientific, Milan, Italy) combined with PC software (Ascent Software, version 2.6).

### 4.11. Cell Viability Assay by Trypan Blue Exclusion Test (TB)

At the end of the different treatments with MAM-2201, the spheroids were washed with PBS (200 μL/well) and dissociated with a trypsin solution (200 μL/well for 5 min at 37 °C); then, a single spheroid was transferred to a microcentrifuge tube. Then, the enzymatic reaction was inactivated with the complete medium, reaching a final volume of 1 mL. The spheroid disaggregation was obtained by carefully pipetting up and down to obtain a single cell suspension, which was then mixed with a 0.4% trypan blue solution (in a ratio of 1:10) for counting by using the Burker chamber in order to determine cell viability. The number of viable cells was determined by light microscopy as a percentage of untreated control cells.

### 4.12. Caspase-3/7 Activity

After the MAM-2201 treatments, the caspase-3/7 activity was evaluated in astrocyte spheroids according to the assay protocol. The luminescence signal was quantified using a microplate fluorometer combined with PC software. The background luminescence (blank) associated with the culture medium used for spheroids was also determined. Then, the experimental values were obtained by subtracting the blank value.

### 4.13. Weight, Diameter, and Mass Density Evaluation by W8 Physical Cytometry

After each exposure time point, the culture medium was carefully removed from each well and n = 13 single D384 spheroids for each time point and concentration were pooled in a microcentrifuge tube. The spheroids were washed twice with PBS (1 mL/tube) and fixed in 4% PF (500 μL/tube) overnight at +4 °C in the dark. The next day, the PF was removed and the spheroids were washed twice and stored at +4 °C until the analysis with the W8 Physical Cytometer (CellDynamics, Bologna, Italy).

Before the measurements, the spheroids were washed twice, moved into a 15 mL centrifuge tube, and resuspended in 7.0 mL of the W8 Analysis Solution (WAS, CellDynamics, Bologna, Italy).

The samples were then analyzed in terms of their mass density (fg/µm^3^), size (diameter—µm), and weight (ng) according to the method of Cristaldi et al. [110].

### 4.14. Data Analyses

Data on the cytotoxicity effects (cell viability, TB, and caspase-3/7 activity) were expressed as the means ± S.E. of three separate experiments, each carried out in three or six replicates. Statistical analyses were performed using one-way ANOVA followed by Tukey’s post hoc test. *p*-values less than 0.05 were considered to be significant.

For the biophysical characterization, a Shapiro–Wilk test followed by Tukey’s method (K > 1.5) were performed to verify the Gaussian distribution of the obtained mass density, weight, and diameter dataset [110]. Statistical analyses were performed using a two-tailed unpaired Student’s *t*-test. The cut-off for significance is indicated in the figure legend.

## 5. Conclusions

The relevance of these findings is related to the consumption of SCs, their consequences on human health, and the continuous placing of new SCs on the market, as well as the increasing evidence that modifications to astrocytes and other glial cells are involved in CNS disorders and neurodegeneration. The use of a suitable cellular model for the screening of neurotoxicity, by applying human-derived CNS cells in three dimensions, can provide an additional valuable tool, mimicking the physiological and biochemical properties of brain tissues better than the traditional two-dimensional in vitro systems, to understand the mechanistic basis of molecular and cellular alterations in the brain. Moreover, the 3D spheroid model allows for the detection of effects caused not only in the short-term, but also for even longer (and repeated) treatment, more likely simulating the different patterns of consumption (e.g., occasional vs. chronic). It may also address questions relating to the examination of long-lasting toxicity effects, which may be derived from an acute intoxication.

## Figures and Tables

**Table 1:** E-cadherin evaluation in D384 spheroids after 24 and 48 h of exposure to MAM-2201.

E-Cadherin (% of Control) in Cell Lysate		
	24 h	48 h
MAM-2201 (µM)		
0	100.00 ± 2.72	100.00 ± 5.26
10	94.63 ± 8.82	100.70 ± 10.57
20	105.46 ± 2.16	72.47 ± 6.30 *
30	102.64 ± 2.94	68.99 ± 5.53 *

Data are expressed as percentages (%) of control (in 10^6^ cells ~1 ng/mL E-Cad) and showed as means ± S.D. of the data obtained from three independent experiments, each performed in duplicate. * *p* < 0.05, statistical analysis by one-way ANOVA followed by Tukey’s multiple comparisons test.

## Data Availability

The datasets used and/or analyzed during the current study are available from the corresponding author upon reasonable request.

## Supplementary Materials

The following supporting information can be downloaded at: https://www.mdpi.com/article/10.3390/ijms24021421/s1.
