# Supplementary material for: Human Astrocyte Spheroids as Suitable In Vitro Screening Model to Evaluate Synthetic Cannabinoid MAM2201-Induced Effects on CNS
Source: Int J Mol Sci. 2023 Jan 11;24(2):1421. doi: 10.3390/ijms24021421 (PMC9861655; doi:10.3390/ijms24021421)
Supplement: Supplementary file 1 [file ijms-24-01421-s001.zip › ijms-2111006-supplementary.pdf]

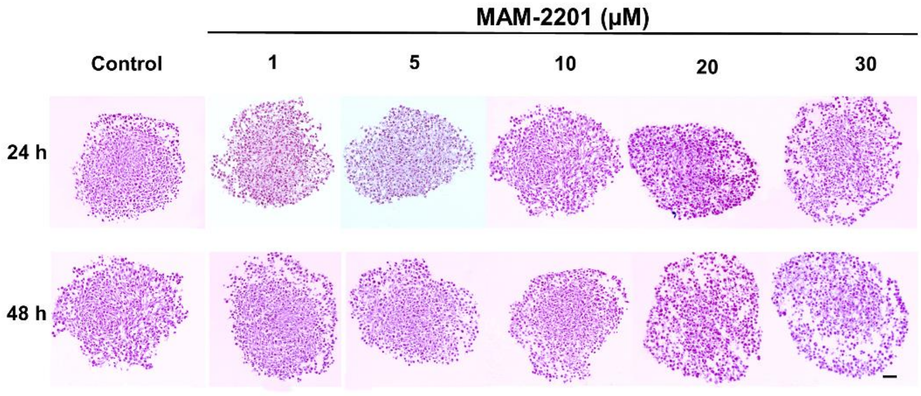

Figure S1. Spheroid histology. The layer of cells was arranged close together and appeared densely packed at the starting density of 200 cells/well in control spheroids samples and in MAM-2201-treated spheroids at the lower concentration tested (1-5 μM) at both 24 and 48 h. No necrotic core presence was observed in both control and treated spheroids. In MAM-2201-treated spheroids a loosely cell packing and poor internal cohesion of cell-to-cell contacts were observed starting from 20 μM after 24 h, and 10 μM after 48 h. Marked presence of interstitial spaces between individual cells was evidenced at the highest concentrations tested (30 μM). Scale bar 100 μm.

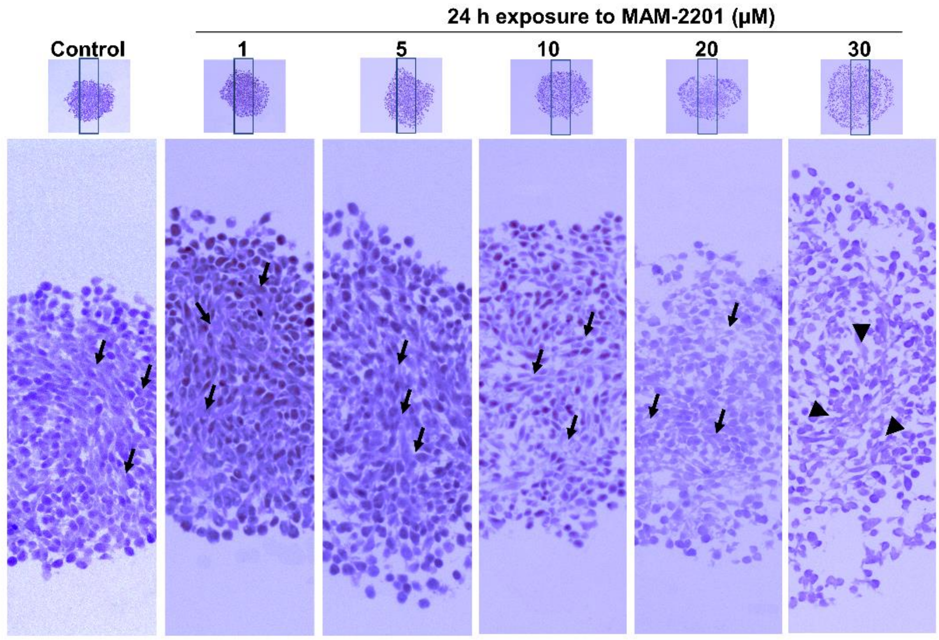

(**a**)

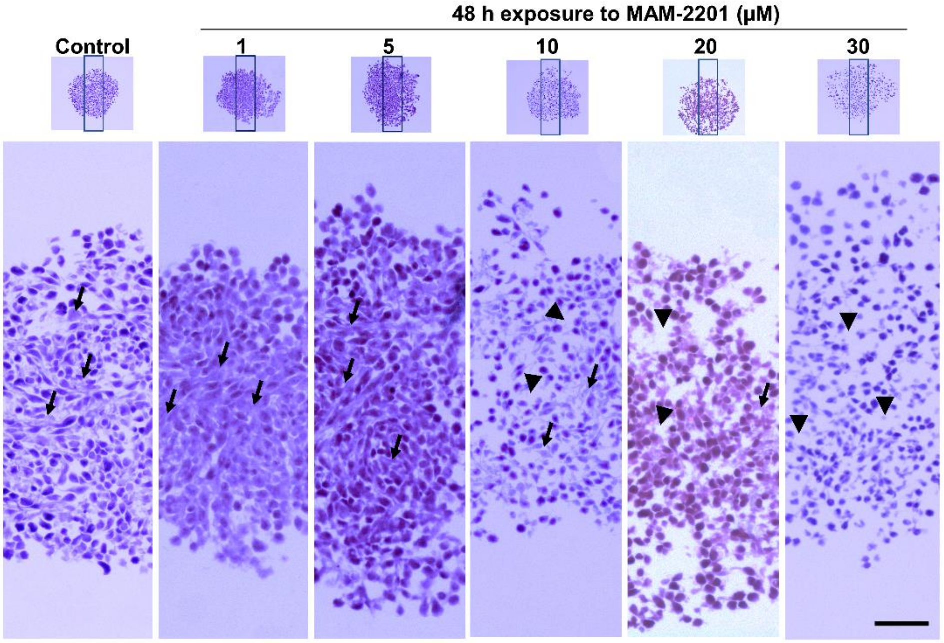

(**b**)

Figure S2. Masson's trichrome staining. Representative images of trichrome staining in D384 spheroid section treated with MAM-2201 (1-30 µM) after 24 h (**a**) and 48 h (**b**). A progressive ECM disassemble started at ≥ 20 µM MAM-2201 after 24 h and ≥ 10 µM MAM-2201 after 48 h: ECM appeared less compact with more empty spaces compared to control. Marked loss of ECM staining was observed at 30 µM MAM-2201 after 24 h with exacerbation after 48 h. Notably, the lower concentration tested (1 and 5 µM) did not affect the ECM staining. Rectangle indicated the magnification (9X) and black arrows indicated the ECM and head indicated the empty spaces (reduction of the amount of collagen fibers). Scale bar 100 µm.
